# Imaging in non-bacterial osteomyelitis in children and adolescents: diagnosis, differential diagnosis and follow-up—an educational review based on a literature survey and own clinical experiences

**DOI:** 10.1186/s13244-021-01059-6

**Published:** 2021-08-09

**Authors:** Matthias C. Schaal, Liya Gendler, Bettina Ammann, Nina Eberhardt, Aleš Janda, Henner Morbach, Kassa Darge, Hermann Girschick, Meinrad Beer

**Affiliations:** 1 Department of Diagnostic and Interventional Radiology, University Hospital Ulm, Albert-Einstein-Allee 23, 89081 Ulm, Germany; 2 Department of Radiology, Children’s Hospital of Philadelphia, Philadelphia, USA; 3 Center for Radiology, Neu-Ulm I Günzburg, Neu-Ulm, Germany; 4 Department of Nuclear Medicine, University Hospital Ulm, Ulm, Germany; 5 Department of Pediatrics and Adolescent Medicine, University Hospital Ulm, Ulm, Germany; 6 Department of Pediatrics, University Hospital Würzburg, Würzburg, Germany; 7 Department of Pediatrics and Adolescent Medicine, Vivantes Klinikum Im Friedrichshain - Landsberger Allee, Berlin, Germany

**Keywords:** Autoinflammatory bone disorders, Chronic non-bacterial osteomyelitis, Imaging methods, MRI, Whole-body imaging

## Abstract

**Background:**

Chronic non-bacterial osteomyelitis (CNO) is an autoinflammatory bone disorder affecting children and adolescents. Previously classified as a rare disease, recent studies suggest a higher incidence of the disease. CNO may develop into the clinical presentation of chronic recurrent osteomyelitis (CRMO) with high relapse rate and multifocality.

**Main body:**

Diagnosis of CNO/CRMO is often delayed, with implications for disease severity and relapse rate. This can be significantly improved by knowledge of the disease entity and its characteristics. Imaging plays a key role in diagnosis, differential diagnosis and therapy monitoring. Magnetic resonance imaging (MRI) has several advantages compared to other imaging methods and is increasingly applied in clinical studies. Recent studies show that a whole-body (WB) coverage (WB-MRI) without contrast agent administration is a rational approach. This educational review is based on a systematic analysis of international peer-reviewed articles and presents our own clinical experiences. It provides an overview of disease entity, incidence and clinical diagnosis. The role of imaging, especially of whole-body MRI, is discussed in detail. Finally, practical advice for imaging, including flowcharts explaining when and how to apply imaging, is provided.

**Conclusion:**

Knowing the specifics of CNO/CRMO and the importance of MRI/whole-body MRI allows rapid and efficient diagnosis as well as therapy support and helps to avoid irreversible secondary damage.

## Key points

- CNO/CRMO is a chronic auto-inflammatory disease of the skeletal system.
- Imaging plays an essential role in diagnosis, differential diagnosis and therapy monitoring.
- MRI/whole-body MRI can be used as a standard for rapid assessment of disease activity.

## Background

### Disease entity, incidence and clinical significance

Chronic non-bacterial osteomyelitis (CNO, often synonymous with non-bacterial osteitis, NBO) is a chronic autoinflammatory disease. The exact etiology is currently unknown. Giedion first described the disease entity in 1972 [1]. Later, the definition of CNO was extended to include chronic recurrent multifocal osteomyelitis (CRMO) [2, 3]. Imaging has played an important role since the time of first description (see [4] for an overview). The incidence of CNO is assumed low with, e.g., 0.4 per 100,000 children per year in Germany [5]. However, in spite of therapy, the relapse rate of symptoms with unfavorable outcomes is high [6]. Increasing awareness of the disease may shorten time to diagnosis [7]. The typical age of first manifestation is 9 to 11 years [5, 7, 8]. The disease is extremely unusual in infancy. Girls are more frequently affected than boys, and there seems to be no difference in ethnicity. An association with the so-called SAPHO syndrome (synovitis, acne, pustulose, hyperostosis, osteitis) has been suspected for many years. A recently published study [9] suggests a similar disease entity, incidence and course of both diseases. On the other hand, low current awareness of the disease might lead to considerable delays between the onset of symptoms and medical diagnosis, which is typically given as 2 years for children [10, 11]. Attempts should be made to diagnose the disease as early as possible, since early therapy can lead to better chances of recovery [12].

*Keep in mind/key point: CNO/CRMO—might be unreported—delay in therapy*

From the clinical point of view, CNO is an important differential diagnosis, which should not be missed. Traditionally, diagnosis of CNO is made by combining assessment of clinical symptoms, laboratory changes, and imaging, with biopsy often performed to exclude bacterial osteomyelitis or malignancy. Clinical symptoms are non-specific and associated with regional pain (mainly continuous and after exertion) and occasional accompanying swelling. Clinical symptoms manifest in a typical distribution of affected body regions and bones. In particular, the long tubular bones of the lower extremity, the vertebral bodies, the clavicles and the mandibula are most commonly affected. Symmetric involvement is considered characteristic for CNO. However, studies show that the disease can also be asymmetric [13]. Changes in laboratory values (like C-reactive protein (CrP) or blood sedimentation rate (BSG)) are variable and can be raised or normal [14, 21].

Historically, *conventional radiographs* and *bone scintigraphy* have played the leading role in diagnostic imaging. Due to its low sensitivity (up to 80% false negative results), conventional radiographs are of only minor utility [15]. Advantages of scintigraphy (technetium-99m-labeled phosphates) include a whole-body coverage, the possibility of suggesting other benign diagnoses, and excluding malignant bone disease [16]. However, it is limited secondary to symmetric physiologic tracer uptake at the physes [17] in children and adolescents, when visualizing osteoblastic activity. *Positron emission tomography* (*PET)* is rarely performed, however may be able to differentiate between chronic and acute disease activity [18]. *Computed tomography* (CT) is only used in individual cases or with older patients, especially to clarify changes in the sternoclavicular joints. [15]. Presently, *magnetic resonance imaging (MRI),* and in particular *whole-body MRI*, is the current imaging modality of choice for CNO imaging in children and adolescents [19]. MRI was initially performed in affected body sites in cases of suspected CNO/CRMO [30]. The first study on whole-body MRI was carried out in 2009 [25], which showed multifocality in almost all patients with superior sensitivity and specificity over clinical examination.

The advantages of MRI include: a noninvasive examination, the absence of ionizing radiation and the possibility of whole-body imaging. Bone marrow edema, which is typical for CNO, can be visualized by fluid sensitive sequences (short TI inversion recovery, STIR or turbo inversion magnitude, TIRM) without the administration of contrast agent. MRI imaging also shows asymptomatic bone changes, which may develop clinical symptoms as the disease progresses.

*Keep in mind/Key point: Diagnostic triad clinic* +* laboratory* + *imaging (especially MRI)*

The increasing importance of CNO/CRMO and the role of imaging in the diagnosis of CNO/CRMO is reflected in the literature. PubMed contains more than 100 publications under the keyword "CNO Imaging," of which more than 30 can be assigned to 2019/2020. Under the search term "CRMO Imaging," there are more than 300 hits, the majority also in the last few years.

There are several published imaging reviews of CNO in children, mainly showing the aspects of different imaging methods. Our aim was to perform a review focusing on CNO and whole-body MRI.

In the following parts of this educational review, results from a systematic literature search are presented in a systematic review, summarizing characteristics of diagnosis, differential diagnosis and follow-up of CNO regarding MRI (I); then, we present a case series with different examples of CNO from our own cohort as an illustration to the review (II); finally, suggestions of clinical workflows in diagnosis, differential diagnosis and follow-up of CNO/CRMO are presented (III).

## Main text

### Part I: systematic review of the literature

#### Search strategy, eligibility criteria and data extraction

**Fig. 1:**
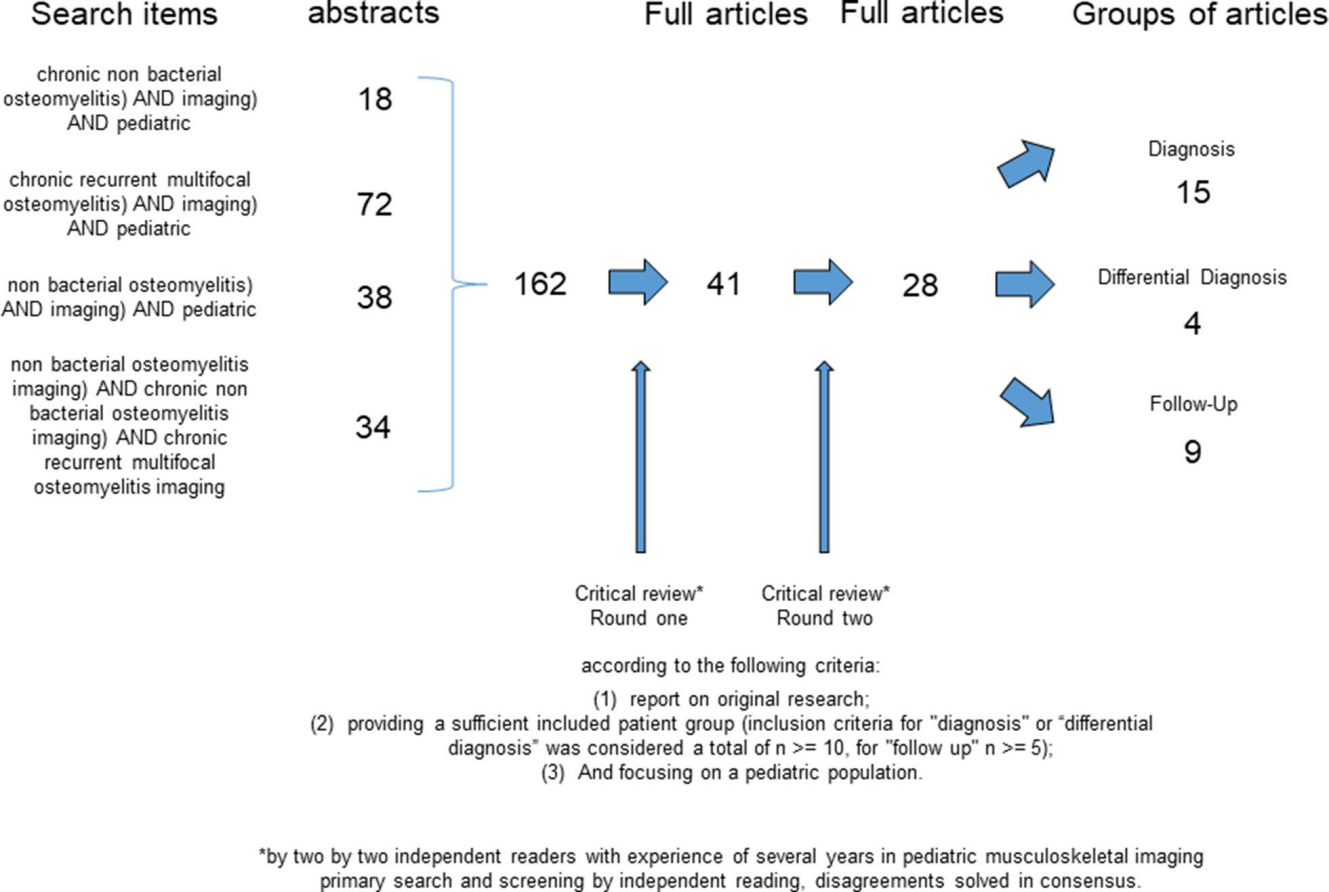
Summary of the comprehensive search strategy as a flowchart

A systematic search of studies in the English language was performed on MEDLINE in September 2020 (PubMed, https://www.ncbi.nlm.nih.gov/pubmed/). The search was limited to original human studies in peer review journals with an available abstract. No publication date limits were applied. Figure 1 summarizes the comprehensive search strategy as a flowchart. Our aim was to identify studies assessing the value of imaging methods (especially MRI) in primary detection, differential diagnosis and follow-up of non-bacterial osteomyelitis in children. Inclusion criteria in detail were: (1) original research; (2) sufficiently large enough patient groups (inclusion criteria for "diagnosis" or “differential diagnosis” were considered a total of *n* ≥ 10, for "follow-up" *n* ≥ 5); (3) focus on a pediatric population; and (4) usage of MRI as the main modality for the assessment of CRMO/CNO.

The search string included the following: “((chronic non bacterial osteomyelitis) AND imaging) AND pediatric" (18 hits) or "((chronic recurrent multifocal osteomyelitis) AND imaging) AND pediatric" (72 hits) or "((non bacterial osteomyelitis) AND imaging) AND pediatric" (38 hits) or "((non bacterial osteomyelitis imaging) AND chronic non bacterial osteomyelitis imaging) AND chronic recurrent multifocal osteomyelitis imaging" (34 hits).

A total of 162 articles were found by using the above search criteria. Initially, article screening was performed by two independent readers (M.S. and M.B. with 5 and 17 years of experience in pediatric musculoskeletal imaging) considering only title and abstract after the removal of duplicates. Both authors read all titles and abstracts independently. All articles that did not meet the inclusion criteria (mainly studies with imaging modalities other than MRI, reviews without original research, or case reports with presentation of a single case) were excluded; the remaining articles were chosen for reading of the full text.

Our primary search and screening process resulted in identification of 41 articles for a full-text review. After independent reading of the full text, articles fulfilling the inclusion criteria (as mentioned above) were selected. Disagreements were resolved by consensus. Finally, references of included articles were hand-searched to check for further eligible studies. The material was again stratified into groups depending on whether primary detection, differential diagnosis or follow-up was addressed in the study.

A total of 28 articles met these final inclusion criteria: 15 articles for "diagnosis," 4 articles for "differential diagnosis" and 9 articles for "follow-up." We hope that with this approach a selection of the most interesting studies was delivered; however, we apologize if we have missed one or more studies.

#### Diagnosis of CNO

All reviewed articles [6, 7, 9, 13, 17, 20–29] concerning the topic “CNO diagnosis” included MRI imaging (see Table 1 for an overview). Mean age of included children was between 10 and 11 years, matching reported ages at primary diagnosis for CNO. 3/15 studies also included adults, one study compared imaging results from children with results from adults.

**Table 1:** Overview for "diagnosis"

Article	Patients with MRI	Patients with WB-MRI	Patients with local MRI	Patients with lesions in MRI	Total lesions in MRI	Lesions per patient in MRI Median	MRI sequences
Roderick et al. [7]	37	30	7	Not specified	162 (incl. x-ray), 47 symptomatic	Not specified	Not specified
Girschick et al. [20]	426 including adults (number not specified)	145	281	Not specified	Not specified	4,1	Not specified
Wipff et al. [21]	40	2	38	Not specified	Not specified	3,1	Not specified
Jansson et al. [13]	72	Not specified	Not specified	Not specified	Not specified	Not specified	not specified
Falip et al. [22]	31	15	Not specified	Not specified	Not specified	Not specified	local MRI: T1-SE, T2-SE fatsat or STIR. WB-MRI: T1-SE, STIR
Padwa et al. [23]	7	None	7	7			FSEIR, T1w, T1w fatsat + C
Ziobrowska-Bech et al. [24]	29	8	21	Not specified	145		Local MRI: T1w, T2w fatsat or STIR. WB-MRI: T1w, STIR
Fritz et al. [25]	13	13		13	101	8	STIR, T1-FSE, T1-FSE fatsat + C
von Kalle et al. [26]	53	53		53	513	8	STIR
Andronikou et al. [27]	37	37		37	317	8,6	STIR, some patients DWI
Leclair et at [28]	16	16		16	33	2	STIR, DWI with ADC
Morbach et al. [17]	32	14	18	32	98 (54 sympt., 44 asympt.)	Not specified	T1-TSE, T1-TSE fatsat + C, TIRM
Skrabl-Baumgartner et al. [9]	24 pediatric, 10 adults	5 pediatric	24 pediatric, 10 adults	Not specified	Not specified	Not specified (all imaging modalities)	Not specified
Arnoldi et al. [29]	40 including adults (number not specified)	17	23	35	112	Not specified	STIR, T1-TSE
Schnabel et al. [6]	48	36	12	48	344	6	Not specified

In most of the studies (13/15), whole-body-MRI was performed in at least in some patients. Ten out of 15 studies included other imaging modalities (mainly conventional radiography, additionally bone scintigraphy in 6 studies, computed tomography in 3 studies). Eight out of 15 studies specified the number of lesions per patient that were detected by MRI, which ranged from 2 to 9 per patient.

All studies that used MRI and other imaging modalities described a significantly higher lesion detection rate with MRI compared to conventional radiography and bone scintigraphy [17]. Most studies reported asymptomatic lesions that were detected only by MRI. This underscores the need and the utility of whole-body MRI in diagnosing CNO.

The site of lesions was specified in 7/15 studies. Only some of the studies that used other imaging modalities mentioned the lesion site in MRI. One study [23] reported only lesions of the mandible; it is known that the lower jaw, along with the temporomandibular joint, can be affected, especially in older children.

CNO lesions show a high predilection for three specific anatomic locations: around the knee (femora, tibiae, fibulae), pelvis (including sacrum and hip) and spine. Bone marrow alterations within the hands and feet can pose a diagnostic dilemma, as physiologic patchy bone marrow signal occur frequently at these locations. Typical patterns of CNO/CMRO involvement in whole-body MRI were first described by von Kalle et al. [26]. These findings also include transphyseal extension with involvement of the metaphysis and epiphysis, periostitis, surrounding soft tissue changes or joint effusion (which may be missed by conventional radiography) [17, 27, 29].

Falip et al. [22] published a longitudinal case series review in 2013 showing many examples of CNO lesions on radiography and MRI. Additionally, a diagnostic strategy was presented as a flowchart based on clinical data, laboratory results and imaging results, focused on whole-body MRI; herein, the authors suggest a therapeutic test with follow-up in patients with results highly suspicious for CNO (multifocal lesions in typical sites such as long bone metaphysis, spine and clavicle, as well as findings of physeal widening). Biopsy was suggested for atypical findings such as solitary lesion or multifocal atypical lesions.

*Keep in mind/Key point: hallmarks of CNO—multifocality—specific pattern with three “hot regions”*

Where specified (9/15) all studies applied STIR or TIRM sequences. Three out of 9 studies included contrast-enhanced series, and 2/9 studies performed Diffusion Weighted Imaging (DWI). Leclair et al. [28] performed whole-body MRI with STIR and diffusion weighted sequences in 16 children with CNO; they reported increased signal within CNO lesions with corresponding increase in the apparent diffusion coefficient (ADC). Diffusion weighting can be helpful in distinguishing between benign inflammatory processes and malignancy.

*Keep in mind/Key point: MRI (ideally WB-MRI) as first-line imaging method*

#### Differential diagnosis of CNO

In view of the clinical symptoms which can wax and wane, and the non-specific laboratory changes, imaging is of great importance, not only to suggest the diagnosis but also to include or exclude other diagnoses from the differential: infection, malignancy and metabolic bone diseases. These include leukemia, lymphoma, Langerhans cell histiocytosis (LCH), osteoid osteoma, osteomyelitis and septic arthritis. In the past, clinical scores or criteria have been developed to separate non-bacterial osteomyelitis from other disease entities, e.g., by Jansson et al. [14].

Only 4 studies were found which met the inclusion criteria for the topic “differential diagnosis” [31–34], Table 2 summarizes the review results. Three of 4 studies were performed as single-center studies; therefore, data are presented in a descriptive way.

**Table 2:** Overview for "differential diagnosis"

Article	Single-center or multicenter	Numbers of WB-MRI	Non-oncologic indication	MRI sequences
Greer [31]	Single-center	360 patients (within 1 year)	250 (69%)	CRMO: coronal STIR, suppl. sag STIR, opt. ax T1-W FSE and T2-W FSE FS or STIR
Schooler et al. [32]	Multicenter	Not specified	Not specified	Coronal STIR (54 of 62 responses, 90%)
Merlini et al. [33]	Single center	54 patients (within 2 years)	24 (44%)	3D STIR
Korchi et al. [34]	Single center	42 patients (within 5 years)	Only non-oncologic	Coronal T1w, coronal 3D SPACE IR, axial STIR-EPI DWI

The age of included patients was—where specified (2/4)—younger than in the groups for “diagnosis” and “follow-up”. Only one study included a large number of patients [31], and the majority of studies included non-oncologic indications. Differential diagnoses were arthritis, neurofibromatosis, pyrexia of unknown origin, myopathy, non-accidental injuries, vasculopathies.

All MRI protocols included STIR imaging, partly accompanied by contrast-enhanced T1-weighted imaging and DWI. While earlier publications saw the administration of MRI contrast agents as a useful supplement particularly in differentiating between malignant diseases, more recent studies have shown that non-contrast MRI examination might be sufficient [35].

Biopsy is recommended in all studies, if results of clinical work-up and imaging are inconclusive. However, systematic studies concerning the optimal time point for the decision toward biopsies are still missing.

*Keep in mind/Key point: sparse data on differential diagnosis and timing of biopsies*

#### Follow-up of CNO

Short- and long-term consequences of CNO often influence quality of life. Among the complications of CNO are sintering vertebral bodies with a resulting kyphosis, which can lead to long-lasting complaints. Not just leg length discrepancies, but more mechanically impactful, angular deformities, which can have profound consequences on gait in the lower extremities. Uncommonly, deformities within the mandible or temporomandibular joint may also occur.

Table 3 summarizes the review results for the topic “follow-up”, which included 9 studies [8, 36–43]. The age range was slightly older than for the topic “diagnosis”. All studies used MRI; 5 studies applied whole-body MRI to all their patients. Only one study included a larger set of patients (*n* = 37), 21/37 with whole-body MRI. Again, STIR or TIRM was used in the majority of studies (8/9) as a standard sequence. In 4/8 studies additionally contrast-enhanced sequences were applied.

**Table 3:** Overview for "follow-up"

Article	Patients with WB-MRI	Patients with follow-up MRI	Time frame between MRIs	Total lesions in initial MRI	Total lesions in follow-up MRI	MRI sequences
Guerin-Pfyffer et al. [36]	9	5	6 months—4 years	28	14	STIR, T1w, T2w
Hofmann et al. [37]	8	8 (1 lost for last follow-up)	After 3 and 6 cycles of Pam, 6 months after last Pam	37	Not specified	T1-TSE, T1-TSE fatsat + C, TIRM
Beck et al. [8]	21	37 (21 WB-MRI)	After 3, 6 and 12 months	184	81 after 12 months	TIRM
Sağ et al. [38]	13	Not specified	Not specified	60	Not specified	Not specified
Miettunen et al. [39]	Only local MRI	9 (local MRI)	Within 4 weeks of symptom recurrence after end of Pam	Not specified, median 3,5	Not specified	T1-SE, T2 fatsat or STIR, T1w fatsat + C,
Moussa et al. [40]		7	Not specified	Not specified	Not specified	local MRI: pre- and post-contrast; WB-MRI: STIR
Gaal et al. [41]	1, rest only local MRI (mandible)	8	Not specified	Not specified	Not specified	T1w, STIR
Roderick et al. [42]	11	11	Before and after Pam therapy, median 16 months	75	32	T1w, STIR, T1w + C
Hospach et al. [43]	Not specified	12 (only with vertebral deformities)	Pam (7 patients): before therapy and after median 13 months	Only specified in patients with Pam	Only specified after Pam	STIR

Monitoring lesion load by imaging (MRI) is important, as different therapeutic agents with varying adverse effect profiles are used for CNO depending on disease severity. In the reviewed studies, nonsteroidal anti-inflammatory drugs (NSAID) were used in all studies, accompanied by pamidronate (8/9), cortisone (5/9) and biologicals (7/9).

The lesion load varied in the individual studies. Where specified (3/9) a reduction >/= 50% was reported. Clinical treatment effects (pain, etc.) were different. In summary, the observed effects under therapy assessed by imaging outweighed the effects assessed by clinical measures/symptoms. Perhaps longer follow-up intervals are necessary to understand the connection between imaging and clinical symptoms.

*Keep in mind/Key point:* There may be discrepancies between changes in lesion load on imaging versus changes in clinical examination.

Typically, NSAIDs are first line therapy. The majority of children show improvement in both imaging and clinical presentation in the first year after therapy [8]. However, longer-follow-up studies show that up to 50% of cases result in a "clinical" relapse in the second year [6].  Second-line therapeutics (in addition to nonsteroidal anti-inflammatory drugs) such as immunosuppressants (methotrexate, TNF-alpha blockers, etc.) and bisphosphonates (which influence bone metabolism) are increasingly used depending to the course of the disease [43].

*Keep in mind/Key point: imaging gate-keeper/decision-support for therapy*

As a noninvasive radiation-free imaging method, MRI is also of particular importance in the assessment of the course of disease in children and adolescents. Complete resolution of osseous abnormalities identified on imaging, with and without treatment, has been described [35]. Deteriorations occurring despite therapy can also be detected by MRI [44].

*Keep in mind/Key point: MRI is an integral part in therapy monitoring*

Selecting the optimal interval for follow-up imaging (MRI) is challenging in daily practice. Typically, the intervals in follow-up were 6 months, sometimes shorter (3 months). One study reported a very wide range of follow-up intervals starting from 6 months up to 4 years [36]. Whole-body MRI is an essential marker for disease activity, as current studies from 2019 show [45, 46]. Standardized radiological follow-up using whole-body MRI is extremely important in order to detect non-responders as early as possible, as relapses can occur early [35]. Even the detection of asymptomatic disease activity by whole-body MRI might hold prognostic value, as recently demonstrated in a study with long-term follow-up examinations [30].

*Keep in mind/Key point: high relapse rate—rationale for early follow-up*

### Part II: presentation of own imaging experiences

#### Case load and imaging examples

Our own clinical experience in CNO imaging encompasses more than 20 children with (partially histological proven) CNO, who are regularly seen in our pediatric outpatient clinic. Additionally, more than 10 children per year with suspected CNO are sent for a secondary opinion. For imaging, ultrasound (to assess the abdominal organs as well as for joint effusion) and MRI, predominantly as whole-body MRI, are used at our institution. Regional MRI is rarely applied in follow-up examinations (see flowchart in part III—Fig. 7). MRI examinations encompass coronal TIRM sequences from head/neck to feet, the latter in axial orientation. Sagittal sequences of the spine are useful. Additionally, T1-weighted sequences are applied in selected body regions (pelvis and spine) to further assess bone marrow changes. Contrast-enhanced imaging is reserved for follow-up in unclear cases. Follow-up intervals are typically every 6 months.

**Fig. 2:**
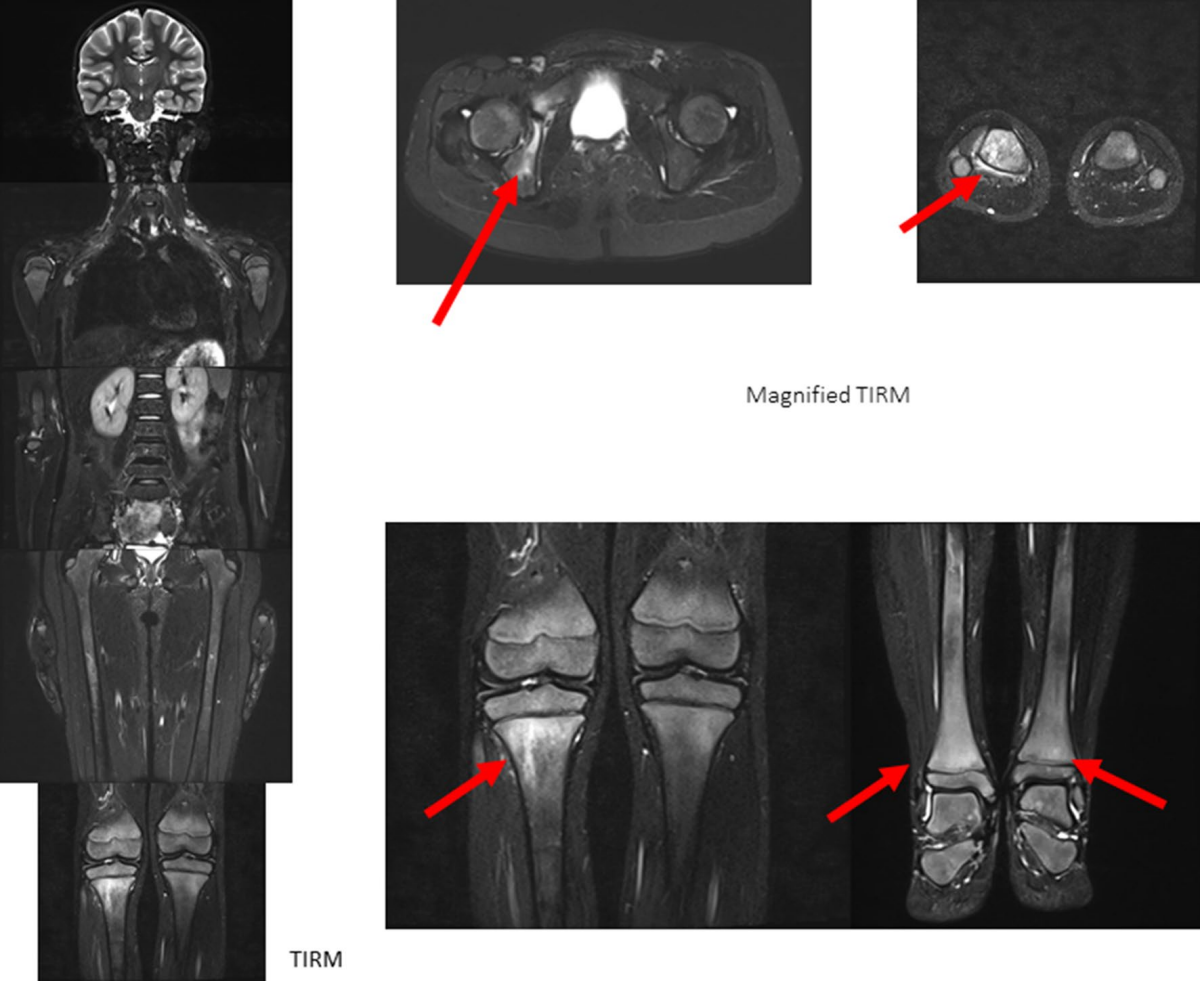
An 8-year-old girl without previous history of MSK pathologies and pain at multiple body regions (back, lower extremities). MRI shows bone marrow edema, affecting the long bones (with focus on epiphyseal and metaphyseal areas) and pelvis (acetabulum)

Figure 2 shows a typical example of primary diagnosis of CNO involvement, affecting the long bones (with focus on epiphyseal and metaphyseal areas) and pelvis (acetabulum) in an 8-year-old girl without previous history of musculoskeletal pathologies. The bilateral involvement is typical, as well as the predominance of juxtaphyseal regions. Coronal views allow a quick and easy understandable overview of disease burden; additional transversal (or sagittal for the spine) images facilitate the quantification of disease burden and also of the possible involvement of paraosseous regions.

**Fig. 3:**
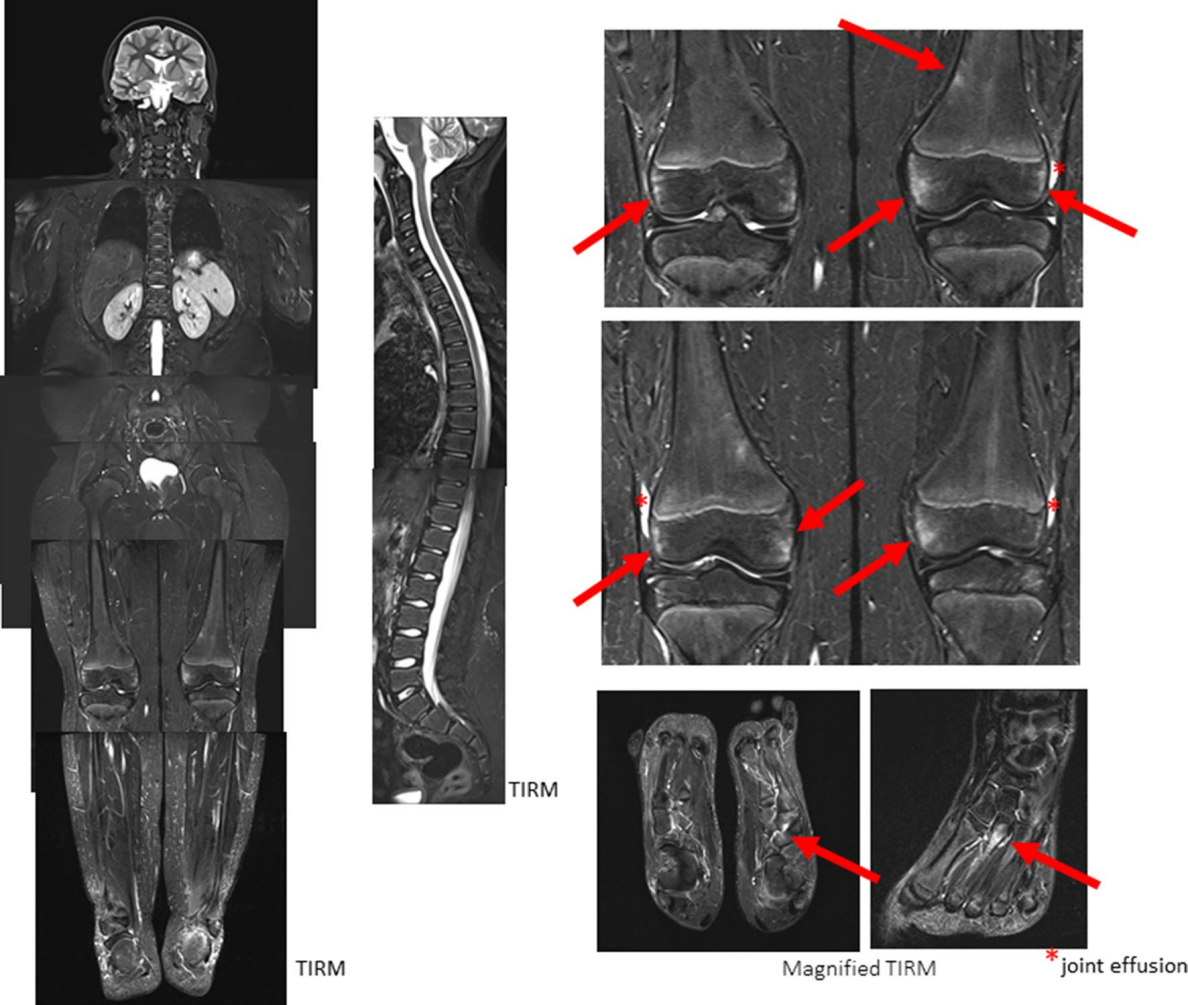
An 11-year-old boy also without previous history of MSK pathologies and pain at multiple body regions (lower extremities, especially knees and feet). MRI shows bone marrow edema—more subtle than in Fig. 1, affecting the long bones (with focus on epiphyseal and metaphyseal areas) and MT

The alterations in the second example of primary CNO diagnosis (Fig. 3) are more subtle. Here, in an 11-year-old boy, patchy bone marrow signal alterations around the knee can be seen accompanied by changes in the second metatarsal bone on the right side and left lateral feet. Other areas of typical CNO involvement (pelvis, spine) is without pathologic alterations. A slight joint effusion of both knees is partly detectable.

*Keep in mind/key point: the extent of lesions (size, signal intensity) varies between individuals*

**Fig. 4:**
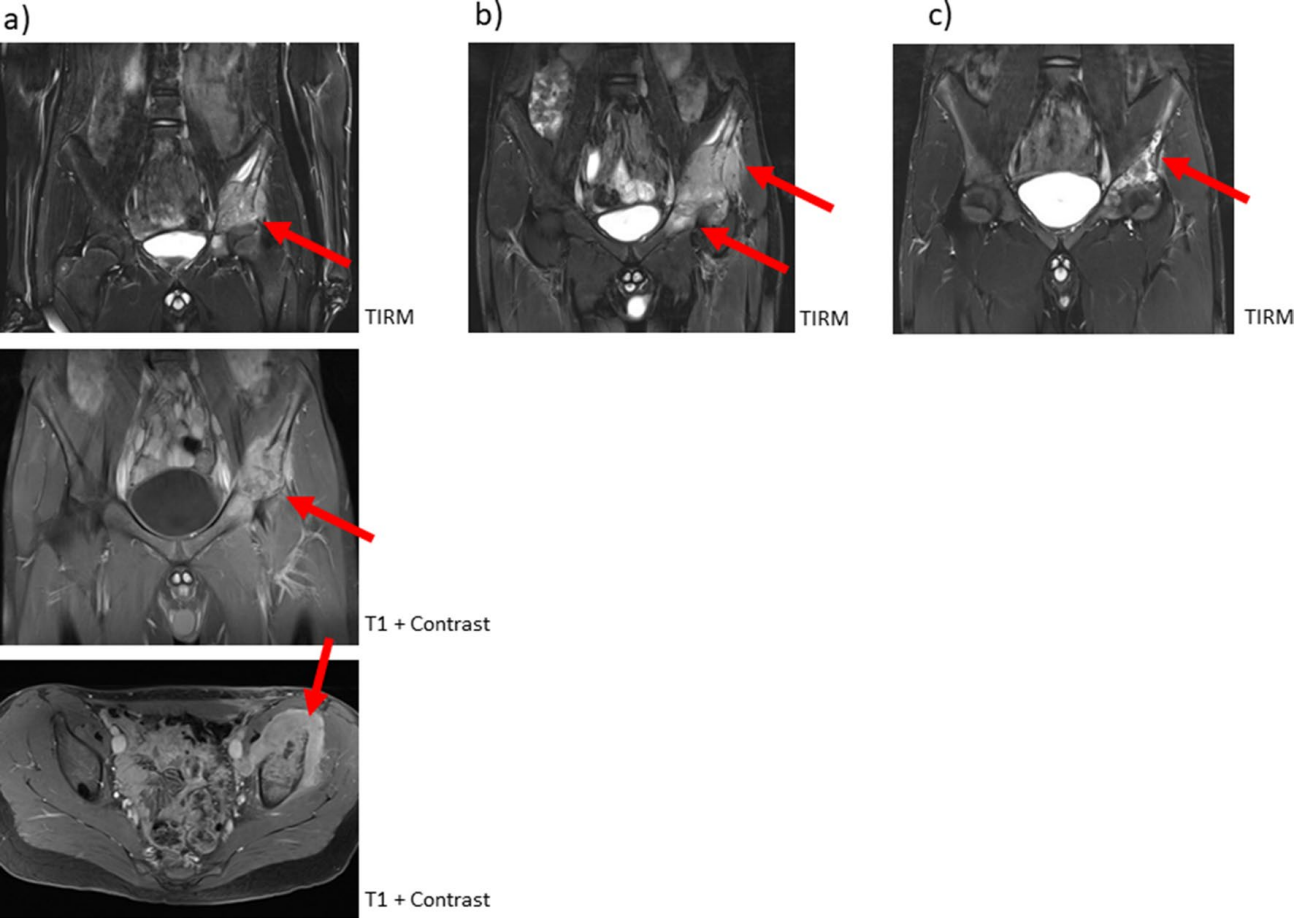
A 14-year-old boy with pain in the left groin and feet. MRI showed an extended focus in the left os ileum/acetabulum with marked paraosseous soft tissue involvement. After bone biopsy of the left os ileum with negative cultures and no evidence of malignancy, therapy was initiated with naproxen, steroids and methotrexate. Three months after the initial MRI, the boy had increasing pain. A follow-up study showed progressive soft tissue swelling with destruction of the left acetabulum/os ileum. After repeat bone biopsy further methotrexate plus pamidronate treatment. A second follow-up MRI showed decreased soft tissue swelling with slightly persisting signal abnormality within the bones

Figure 4 shows an example of the difficulties in differential diagnosis of CNO diagnosis. A 14-year-old boy complained about left-sided pelvic pain persisting for several weeks. MRI (TIRM and contrast-enhanced T1w) demonstrates a huge mass in the left os ilium with diffuse infiltration of the paraosseous tissue (Fig. 4a). Bone biopsy showed neither malignant cells, nor bacterial involvement. Therapy with NSAID, corticosteroids and methotrexate were started for the presumed diagnosis of CNO. As clinical symptoms did not improve, MRI was repeated after 3 months, which also showed no improvement (Fig. 4b). Biopsy was repeated and showed the same histopathology, most compatible with CNO. Therapy was supplemented by pamidronate with resolution of clinical symptoms and improvement on imaging (10 months later, Fig. 4c). This example demonstrates that, in some cases only, repeat histology, as well as follow-up MRI, allows an exact diagnosis and exclusion of malignant disease.

*Keep in mind/key point: DD might be difficult and might include biopsy plus imaging follow-up*

**Fig. 5:**
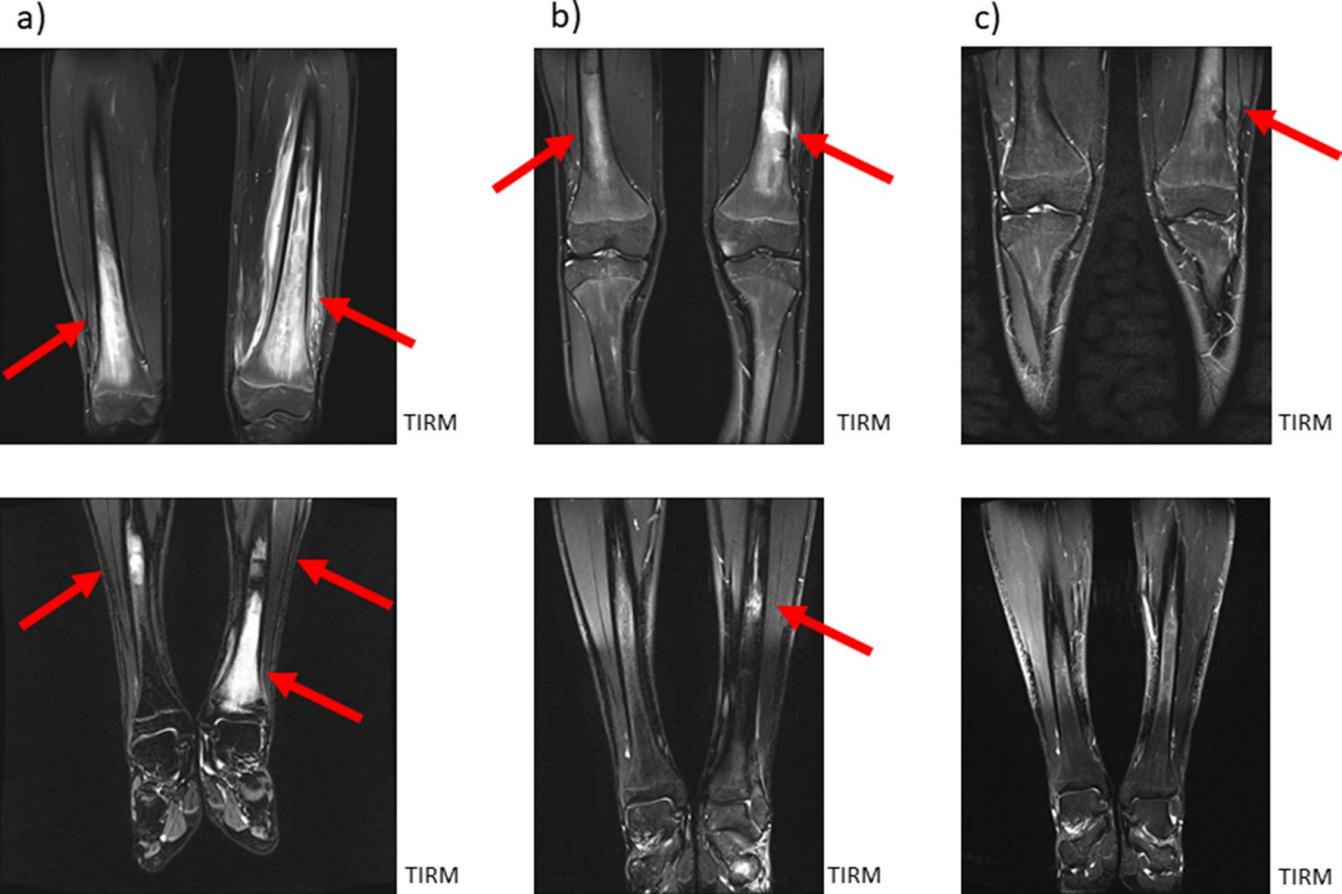
A 16-year-old adolescent with exhaustion for 2 months, bone and muscle pain. MRI showed extensive bone marrow edema in the distal femora and tibiae, marked concomitant soft tissue reaction within the left femur and less marked in the left tibia. After bone biopsy (femur) and diagnosis of CNO, initial therapy with naproxen yields clinical regression. MRI demonstrated regression. Under naproxen therapy asymptomatic patient with still visible lesions

**Fig. 6:**
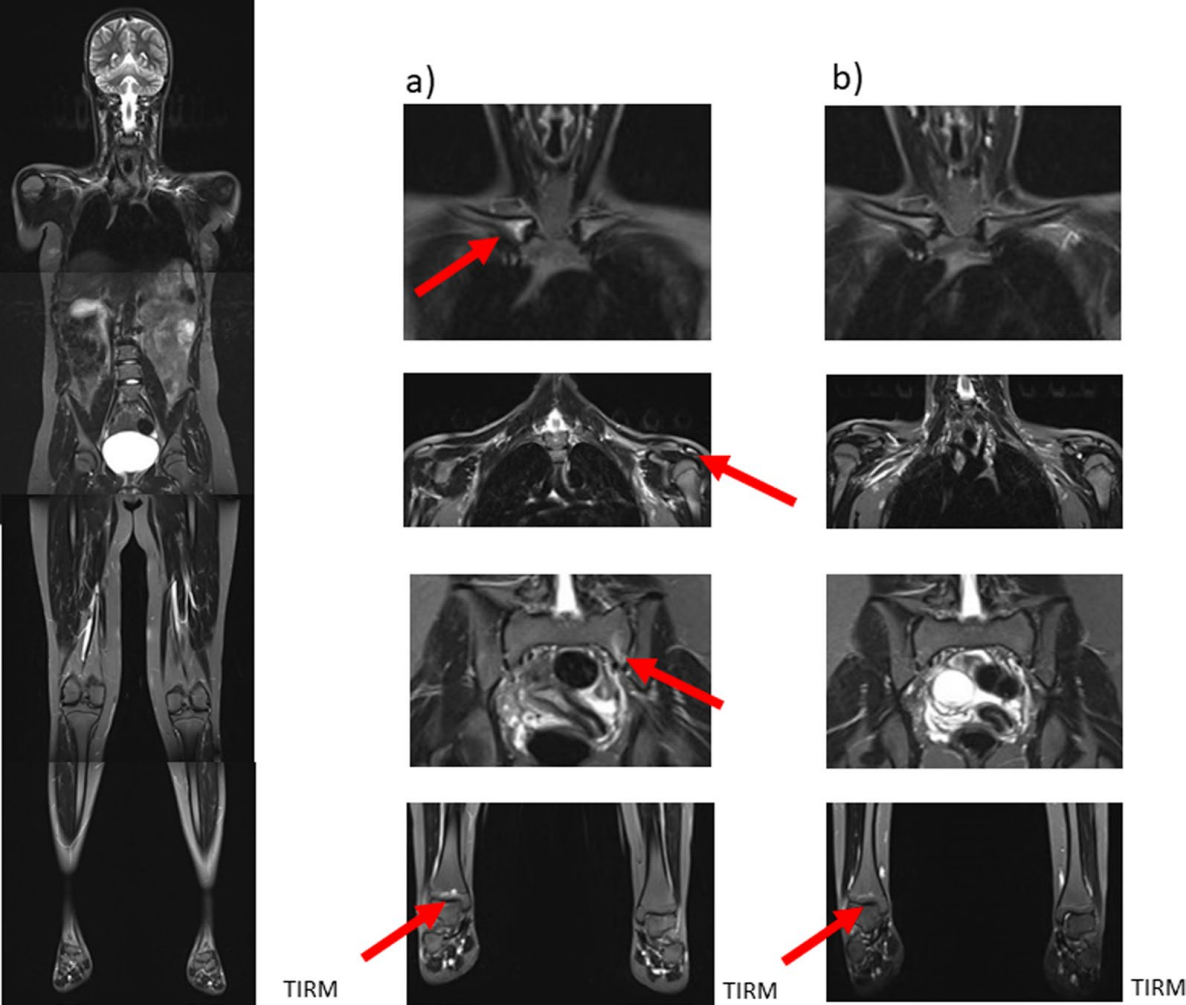
A 12-year-old girl, multifocal CNO. Whole-body MRI (left column) with (foci in the right clavicle, left acromion, left pars lateralis ossis sacri and distal right tibia (enlargements in the middle column). After therapy with naproxen and pamidronate, subtotal regression of the foci (right column)

Figures 5 and 6 show examples of follow-up of CNO involvement.

Impressive bone marrow alterations were seen in an older male (16 years) boy with history of acne, pustulosis and joint effusions, accompanied by bone pain. MRI detected signal abnormalities predominantly located within the long bone diaphyses of the lower extremities (Fig. 5a). Follow-up after initiation of naproxen showed regression of the osseous alterations (after 6 months, Fig. 5b) with persistence of bone marrow changes on the second post-treatment scan despite of complete clinical regression (after 18 months, Fig. 5c).

Disease burden and degree of changes under therapy are considerably more subtle in the second example of follow-up (Fig. 6). Here, in a 12-year-old girl, bone marrow alterations are seen in the right medial clavicle, the left acromion (paraosseous), the left massa lateralis of the sacrum and the right lower tibial epiphysis (Fig. 6a). Subtotal regression of lesions can be detected in follow-up MRI under therapy with naproxen and pamidronate (after 8 months, Fig. 6b).

*Keep in mind/key point: bone marrow changes might persist even in complete clinical remission*

### Part III: conclusions with a synopsis and practical advice

It is well known that chronic non-bacterial osteomyelitis is difficult to assess [47, 48] and that an elevated awareness of this disease is essential to appropriately identify and manage patients [49]. Our aim was to perform a review focusing on CNO and whole-body MRI with special emphasis on the clinical settings of primary diagnosis, differential diagnosis and therapy monitoring. As demonstrated by this review, non-contrast whole-body MRI represents an essential pillar of detection and treatment of CNO.

**Fig. 7:**
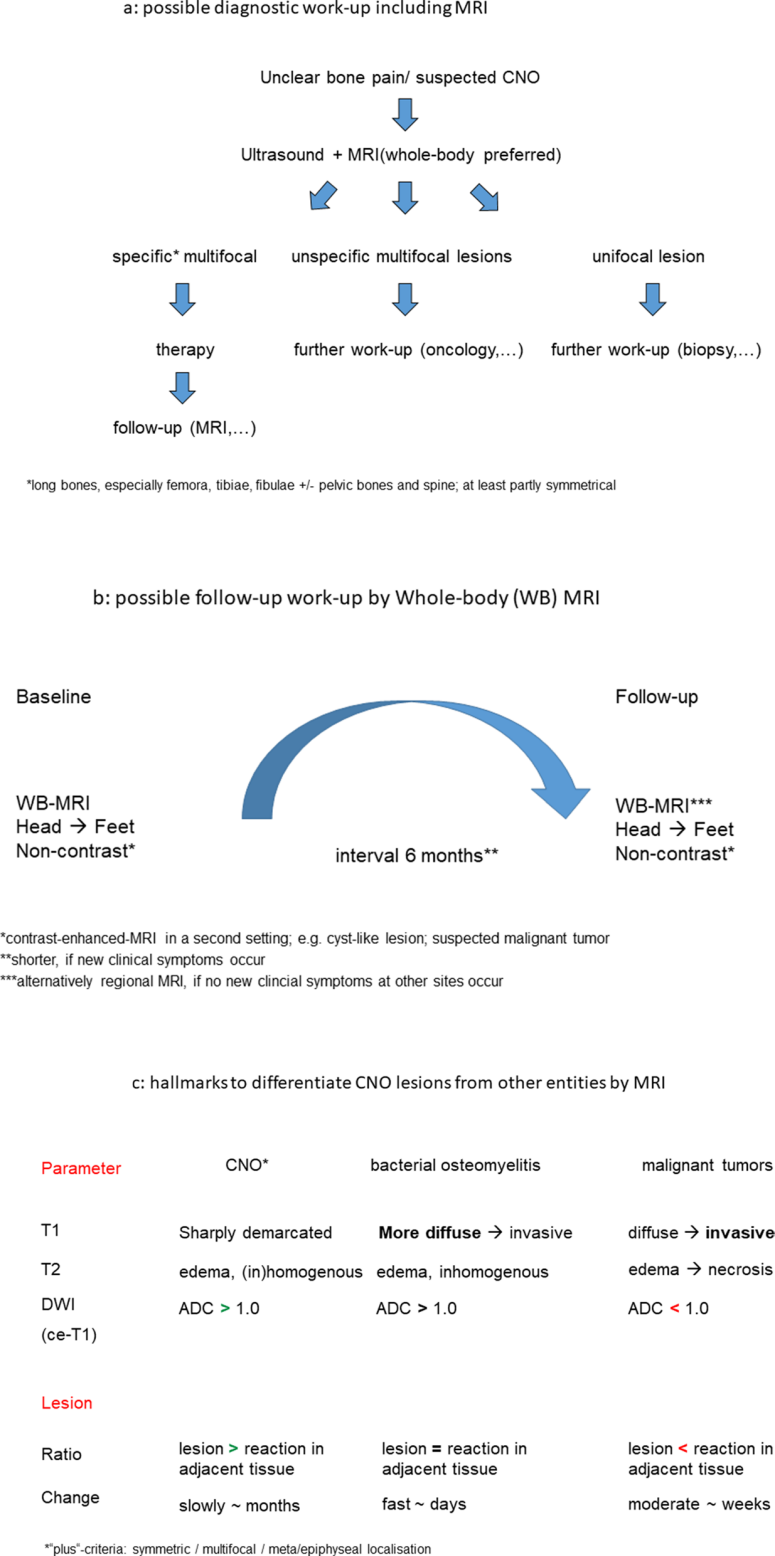
Flowcharts for diagnostic work-up (**a**), MRI algorithms in primary diagnosis and follow-up (**b**) and imaging hallmarks for differential diagnosis between non-bacterial (CNO), bacterial osteomyelitis and malignant bone lesions (**c**)

However, as the reviewed algorithms concerning usage of imaging methods and time intervals in disease/treatment follow-up currently vary from center to center, standardized flowcharts might be helpful to improve detection of CNO, to reduce the number of multimodality imaging examinations and to facilitate the communication between referring physicians and radiologists. Therefore, we developed three flowcharts, which summarize a possible diagnostic work-up (Fig. 7a), a possible MRI algorithm in primary diagnosis and follow-up (Fig. 7b) and—finally—imaging hallmarks, which helps difference between non-bacterial (CNO), bacterial osteomyelitis and malignant bone lesions (Fig. 7c).

### Outlook

In the coming years, progressive understanding of disease pathophysiology, treatment options and, last but not least, imaging methodology is to be expected. First of all, the results of recently initiated multicenter trials will shed light on the advantages as well as current limitations of whole-body MRI examinations [50]. Additionally, atlases and textbooks will soon be available, which will allow a systematic overview, especially in whole-body MRI imaging of CNO bone lesions. Progress is also expected in the development of a uniform, globally accepted scoring system, with a first approach recently published [51]. Finally, support in image post-processing and/or reporting by artificial intelligence (AI)-driven segmentation or decision-support devices [52] will facilitate CNO detection and quantification in daily routine.

## Data Availability

Not applicable, this is an educational review.

## Abbreviations

ADC: Apparent diffusion coefficient
BSG: Blood sedimentation rate
CNO: Chronic non-bacterial osteomyelitis
CRMO: Chronic recurrent multifocal osteomyelitis
CrP: C-reactive protein
CT: Computed tomography
DXA: Dual energy X-ray absorptiometry
F-18-FDG: F-18-fluorodeoxyglucose
LCH: Langerhans cell histiocytosis
MRI: Magnetic resonance imaging
NBO: Non-bacterial osteitis
SAPHO syndrome: Synovitis, acne, pustulose, hyperostosis, osteitis
